# Propensity of US Military Personnel to Seek Mental Health Care When Community Psychiatric Capacity Changes

**DOI:** 10.1001/jamahealthforum.2023.3330

**Published:** 2023-10-06

**Authors:** Yu-Chu Shen, Marigee Bacolod, Jennifer A. Heissel

**Affiliations:** 1 Department of Defense Management, Naval Postgraduate School, Monterey, California; 2 National Bureau of Economic Research, Cambridge, Massachusetts; 3 IZA Institute of Labor Economics, Bonn, Germany

## Abstract

**Importance:**

Understanding how the active duty military population’s mental health care use is associated with local military and civilian psychiatrist capacity is critical in designing the optimal allocation of mental health resources from both sectors to improve the mental health of military personnel.

**Objective:**

To evaluate whether the probability of mental health care visits by military personnel changes when psychiatrist capacity changes in their communities, when capacity is measured separately for military treatment facilities and civilian sectors.

**Design, Setting, and Participants:**

This cohort study of active duty US military service members between January 1, 2016, and September 30, 2020, combines data from the Defense Health Agency, the National Plan and Provider Enumeration System, and the US Census. Data were collected and analyzed from June 2022 to July 2023.

**Main Outcomes and Measures:**

The main outcome was the probability of making at least 1 mental health care visit in a given quarter at military treatment facilities and in civilian settings. Linear probability models with 2-dimensional fixed effects at individual and community levels were implemented to estimate changes in individual outcomes when community psychiatrist capacity changed.

**Results:**

This study includes 1 958 421 US service members (83% men; mean [SD] age at baseline, 28.4 [8.0] years). Thirteen percent of service members did not have military treatment facility psychiatrists available within a 30-minute driving time, and 66% lived in communities with a psychiatrist shortage (<1 psychiatrist per 20 000 relevant population), while 9% lived in communities with high (>3 psychiatrists per 20 000 relevant population) military treatment facility psychiatrist capacity. Five percent of service members lived in communities with no civilian psychiatrists within a 30-minute driving time, while 66% lived in communities with high civilian psychiatrist capacity. The mean quarterly mental health care visit rates to military treatment facilities and civilian settings were 7% and 2%, respectively. The probability of a mental health care visit to a military treatment facility increased by 0.95 percentage points (95% CI, 0.79-1.10 percentage points; equivalent to 14%) when the individual experienced a change in military treatment facility capacity from no psychiatrist to high capacity. The probability of a mental health care visit to a civilian setting increased by 0.57 percentage points (95% CI, 0.38-0.76 percentage points; equivalent to 32%) when civilian capacity changed from no psychiatrist to high capacity. The magnitude of responses to military treatment facility capacity changes remained similar in communities that already had high civilian capacity.

**Conclusions and Relevance:**

This cohort study of the US military population suggests that active duty military personnel rely largely on military treatment facilities for their mental health care and that there are meaningful responses to military treatment facility psychiatrist capacity changes even in communities with high civilian psychiatric capacity. Realigning military treatment facility psychiatrists across communities with shortages and high-capacity military treatment facilities, as well as addressing nongeographical barriers in the civilian sector, remain critical to achieve the optimal balance between military and civilian care provision.

## Key Points

**Question:**

How does the probability of mental health care visits by military personnel change when psychiatrist capacity changes in their communities, where capacity is measured separately for military treatment facilities and civilian sectors?

**Findings:**

In this cohort study of active duty US military members between 2016 and 2020, three-fourths of all mental health care visits occurred in military treatment facilities. When military treatment facility psychiatrist capacity within a 30-minute driving time changed from zero to high capacity, the probability of mental health care visits to military treatment facilities increased by 0.95 percentage points, and there was no change for visits to civilian psychiatrists; when civilian psychiatrist capacity changed from zero to high, the visit rate to military treatment facilities decreased by 2.58 percentage points, while the visit rate to civilian psychiatrists increased by 0.57 percentage points.

**Meaning:**

This study suggests that realigning military treatment facility psychiatrists across communities with shortages and high-capacity military treatment facilities, as well as addressing nongeographical barriers in the civilian sector, remain critical to achieve the optimal balance between military and civilian care provision.

## Teaser

This cohort study evaluates whether the probability of mental health care visits by military personnel changes when psychiatrist capacity changes in their communities, when capacity is measured separately for military treatment facilities and civilian sectors.

## Introduction

Military service members have higher mental health care needs than civilians, including care for posttraumatic stress disorder (PTSD), depression, and suicidal ideation.^1,2,3^ The higher prevalence and severity of mental health conditions among military service members often require the specialized care of psychiatrists. The military directly provides health services through military treatment facilities and purchases health care services from civilian health care professionals.^4^ TRICARE, the health insurance program of the US military, ensures that all service members have insurance to cover mental health services. Services accessed at a military treatment facility are automatically covered, while civilian mental health care professionals decide whether to accept TRICARE based on reimbursement rates, their capacity, and other factors.^5^ Active duty individuals have $0 copays for care received at military treatment facilities.^6^ Service members also have a $0 copay with a referral and preauthorization from their primary care manager to receive care from a civilian mental health care professional who accepts TRICARE. For comparison, Medicare requires patients to pay 20% of the Medicare-approved cost for mental health care.^7^ Service members accrue costs if they work outside of their primary care manager or visit a mental health care professional who does not accept TRICARE.^8^ The US Department of Veterans Affairs can also provide care to eligible beneficiaries on a space-available basis.^9^

The military population’s higher need for mental health care does not always translate into higher use of mental health care.^10,11,12,13^ Military deployments and frequent relocations make it difficult to maintain consistent access to treatment. Many active duty service members live in places with very limited mental health service capacity. Between January 2016 and September 2020, more than one-third of TRICARE beneficiaries lived in communities with a shortage of both military treatment facilities and civilian psychiatrists.^14^ These limitations translate into long waiting times and difficulty scheduling care.^15^ Furthermore, many active duty individuals cannot take advantage of civilian mental health care capacity because many civilian health care professionals do not accept TRICARE.^16,17^ Last, care-seeking behavior is associated with individual traits. For example, studies of veterans found that PTSD and ease of access to care are common markers of mental health use for both men and women, but depression and low-income status are markers of mental health use for women but not men.^11,12^

Although most past studies provided insights regarding individual traits associated with care-seeking behavior, few focused on how the built environment (ie, mental health resource capacity available in the community) is associated with care-seeking behavior in the military population. One study of non–active duty TRICARE beneficiaries found that beneficiaries in geographical areas with a military treatment facility available as primary care clinics had lower use of primary care but higher use of specialty care than TRICARE beneficiaries in geographical areas that had civilian primary care providers.^18^ On the other hand, TRICARE beneficiaries increased their use of civilian facilities from 2000 to 2014, but they occurred mostly in emergency departments, a costly setting that does not provide continuity of care.^19^ Understanding how the built environment is associated with use of care is critical because it is precisely the built environment where policy levers can be implemented to improve overall mental health care for military personnel.

To fill these gaps, we use longitudinal data that follow all active duty personnel between 2016 and 2020 to address the following question: how does the probability of a mental health care visit by military personnel change when psychiatrist capacity changes in their communities, where capacity is measured separately for military treatment facility and civilian sectors? We separately examined 2 outcomes: the probability of at least 1 mental health care visit to a military treatment facility in a given quarter and the probability of a visit to a civilian psychiatrist. We hypothesized that changes in mental health care visit rates would be positively associated with changes in a community’s psychiatrist capacity. Furthermore, we hypothesized that the response to changes in military treatment facility capacity would be bigger than the response to changes in civilian capacity because there are fewer nongeographical barriers to accessing military treatment facility psychiatrists than civilian psychiatrists.

## Methods

### Study Population and Data

Our study population contained 22 158 647 person-quarters representing 1 958 205 unique service members from the US Army, Navy, Air Force, and Marine Corps who served on active duty at any time between January 1, 2016, and September 30, 2020. We obtained personnel data from the Defense Enrollment Eligibility Reporting System and clinical diagnoses of mental health conditions from all medical visits and visit dates in both military treatment facilities and civilian settings from the Defense Health Agency. The Naval Postgraduate School institutional review board approved this study with a waiver of consent because data were deidentified. The study followed the Strengthening the Reporting of Observational Studies in Epidemiology (STROBE) reporting guideline.

We obtained monthly psychiatrist capacity data from the Medical Expense and Performance Reporting System and the Defense Medical Human Resource System internet for military capacity and the National Plan and Provider Enumeration System National Provider Identifier data for civilian psychiatrists. We used the US Census to obtain the civilian population count at the zip code level to construct civilian psychiatrist capacity measures. We used a web-based query^20^ to geocode and derive a database of driving times between centers of each service member’s community and (1) the military treatment facility and (2) zip code centers of civilian psychiatrists’ practicing location.

### Outcome of Interest

Because more than 92% of service members have zero mental health visits in any given quarter, we examined 2 binary outcomes of whether a service member had at least 1 mental health care visit in a given quarter (1) in military treatment facilities or (2) to civilian psychiatrists. A health care visit was considered mental health related if it contained *International Statistical Classification of Diseases and Related Health Problems, Tenth Revision*, codes F01 to F99.^21^ Examples include diagnoses such as PTSD, bipolar disorder, schizophrenia, and major depression but exclude conditions that are more commonly treated by nonpsychiatrists, such as counseling, maladjustment, and problems related to the social environment.

### Defining Community Psychiatrist Capacity

The key factor associated with probability of a mental health care visit in our statistical model was the psychiatrist capacity within a 30-minute driving time of a service member’s community. Using metrics developed in a prior study (see eAppendix 1 in Supplement 1 for details),^14^ we defined *military treatment facility capacity* as the number of military treatment facility psychiatrists available within a 30-minute driving time divided by the number of TRICARE beneficiaries (because military treatment facilities are not accessible to non-TRICARE residents) within the same set of zip codes.^22^ Similarly, *civilian capacity* was defined as number of civilian psychiatrists divided by number of total residents (including the civilian population) within the same collection of zip codes.

For ease of interpretation and to reduce estimation bias, we converted the linear capacity measure into the following categories, separately for military treatment facilities and civilian facilities: no access (0 psychiatrists within 30 minutes), shortage (<1 psychiatrist per 20 000 relevant population), adequate (1-3 psychiatrists per 20 000 relevant population), and high (>3 psychiatrists per 20 000 relevant population). Communities with no psychiatrists within a 30-minute driving time were the reference group. The cutoff for community shortages is based on the Health Resources and Services Administration definition.^14,23^ The cutoff for a high-capacity community is based on a Department of Health and Human Services report that documented that 3 psychiatrists were needed for every 20 000 people.^24,25^

### Statistical Analysis

Data were collected and analyzed from June 2022 to July 2023. Because our outcomes are binary, we implemented linear probability models with 2-dimensional fixed effects at both individual and community levels and estimated robust SEs that accounted for intraperson correlation across time and interperson correlation from the same community.^26,27^ All individual demographic and time-invariant community characteristics were subsumed by the 2 sets of fixed effects. Due to the large number of fixed effects, logit would not be appropriate because of incidental parameter problems.^28^ The 2-dimensional fixed effects were critical because they removed any time-invariant unobservable characteristics from a given individual (eg, an individual’s underlying mental health need, care-seeking preference, prior exposure to trauma, and family history), while the community fixed effects remove any time-invariant unobservable characteristics across communities (eg, community socioeconomic conditions). Under this empirical framework, the estimated change in an individual’s mental health outcome was associated with 2 sources of variation: (1) when there was within-community capacity change and (2) when a service member moved to a different community. The main variation comes from the service members who moved, as 70% of service members moved at least once during the study period, while the within-community capacity was stable over time, with only 4% of communities switching military capacity categories during the study period and 8% of communities switching civilian capacity categories during the study period.

Additional control variables included time-varying events that could affect a person’s mental health need (whether a person was demoted, divorced, got married, gained a dependent child, moved to a different location, or returned from overseas deployment in prior or current quarter) and indicators for each year-quarter to control for macro trends. All *P* values were from 2-sided tests and results were deemed statistically significant at *P* < .05. All models were estimated using Stata, version 17 (StataCorp LP).^29^

## Results

Table 1 shows the characteristics of the study population (N = 1 958 421; 83% men and 17% women; mean [SD] age at baseline, 28.4 [8.0] years) stratified by service members who had no mental health care visit during the study period (n = 1 312 653) and those with at least 1 visit (n = 645 768). The demographic characteristics reflect the distribution of the US Armed Services (6% Asian or Pacific Islander service members, 16% Black service members, 16% non-White Hispanic service members, 56% White service members, and 5% service members of other or unknown race and ethnicity, including American Indian or Alaska Native service members).

**Table 1.** Descriptive Statistics of Study Population, Overall and by Mental Health Care Visit Status

Statistic	All population, No. (%) (N = 1 958 421 [100])	Never had any mental health visit, No. (%) (n = 1 312 653 [67])	Had at least 1 mental health visit, No. (%) (n = 645 768 [33])	Difference
Had mental health care visit in 2016-2020	645 768 (33)	0	645 768 (100)	NA
Visited military treatment facility only	444 692 (23)	0	444 692 (69)	NA
Visited civilian psychiatrist only	55 248 (3)	0	55 248 (8)	NA
Visited both settings	145 828 (7)	0	145 828 (23)	NA
Service member demographic characteristics				
Sex				
Female	331 044 (17)	194 141 (15)	136 903 (21)	−6^a^
Male	1 627 380 (83)	1 118 512 (85)	508 865 (79)	
Race and ethnicity				
Asian or Pacific Islander	114 711 (6)	81 945 (6)	327 66 (5)	1^a^
Black	322 822 (16)	198 837 (15)	123 985 (19)	−4^a^
Non-White Hispanic	316 195 (16)	221 556 (17)	94 639 (15)	2^a^
White	1 103 428 (56)	739 047 (56)	364 381 (56)	0
Unknown or other race^b^	101 265 (5)	71 268 (5)	29 997 (5)	1^a^
Married	1 072 155 (55)	669 386 (51)	402 769 (62)	−11^a^
Age at baseline, mean (SD), y	28.4 (8.0)	27.5 (7.6)	30.4 (8.4)	−3^a^
Service characteristics				
Army	752 578 (38)	450 438 (34)	302 140 (47)	−12^a^
Navy	464 267 (24)	328 859 (25)	135 408 (21)	4^a^
Air Force	438 724 (22)	307 353 (23)	131 371 (20)	3^a^
Marines	302 852 (15)	226 003 (17)	76 849 (12)	5^a^
Enlisted	1 664 268 (85)	1 087 985 (83)	576 283 (89)	−6^a^
Officer	294 123 (15)	224 640 (17)	69 483 (11)	6^a^
Events that occurred during the study period				
Demoted	85 921 (4)	35 199 (3)	50 722 (8)	−5^a^
Divorced	175 443 (9)	85 632 (7)	89 811 (14)	−7^a^
Married	387 592 (20)	245 943 (19)	141 649 (22)	−3^a^
Gained a nonspouse dependent	448 685 (23)	280 360 (21)	168 325 (26)	−5^a^
Moved to a different community	1 351 774 (69)	917 726 (70)	434 048 (67)	2^a^
Deployed overseas	276 598 (14)	207 571 (16)	69 229 (11)	5^a^

Abbreviation: NA, not applicable.

^a^ *P* < .001.

^b^ Other race includes American Indian or Alaska Native and race and ethnicity groups not identified in the table.

During the study period, 33% of the active duty personnel had at least 1 mental health–related visit to either a military treatment facility or a civilian psychiatrist. Among those who had a mental health care visit, 70% saw psychiatrists only in military treatment facility settings, 9% saw only civilian psychiatrists, and the remaining 21% used both settings. Those who made mental health care visits were more likely to be female (21% among those with ≥1 visit vs 15% among those with no visit) and married (62% among those with ≥1 visit vs 51% among those with no visit). The Army had a disproportionately higher share of service members with at least 1 mental health care visit (47% compared with 34% among the sample with no mental health care visit).

**Figure 1.**
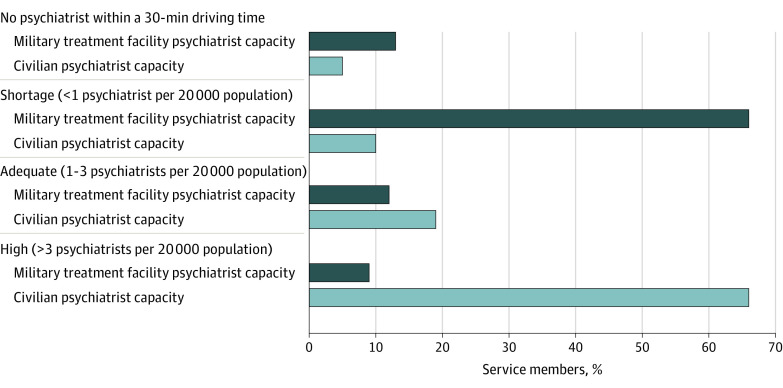
Distribution of Active Duty TRICARE Beneficiaries by Community Psychiatric Capacity Categories Community capacity quartiles are computed separately for military and civilian sectors and are based on the number of psychiatrists per 20 000 relevant population within a 30-minute driving time.

Figure 1 shows the distribution of active duty service members by their community’s psychiatrist capacity. Thirteen percent of service members did not have military treatment facility psychiatrists available within a 30-minute driving time, 66% lived in communities with shortages, and 9% lived in communities with high military treatment facility psychiatrist capacity. For civilian capacity, only 5% of service members lived in communities with no access to a civilian psychiatrist within a 30-minute driving time, while 66% lived in communities with a high civilian psychiatrist capacity.

**Figure 2.**
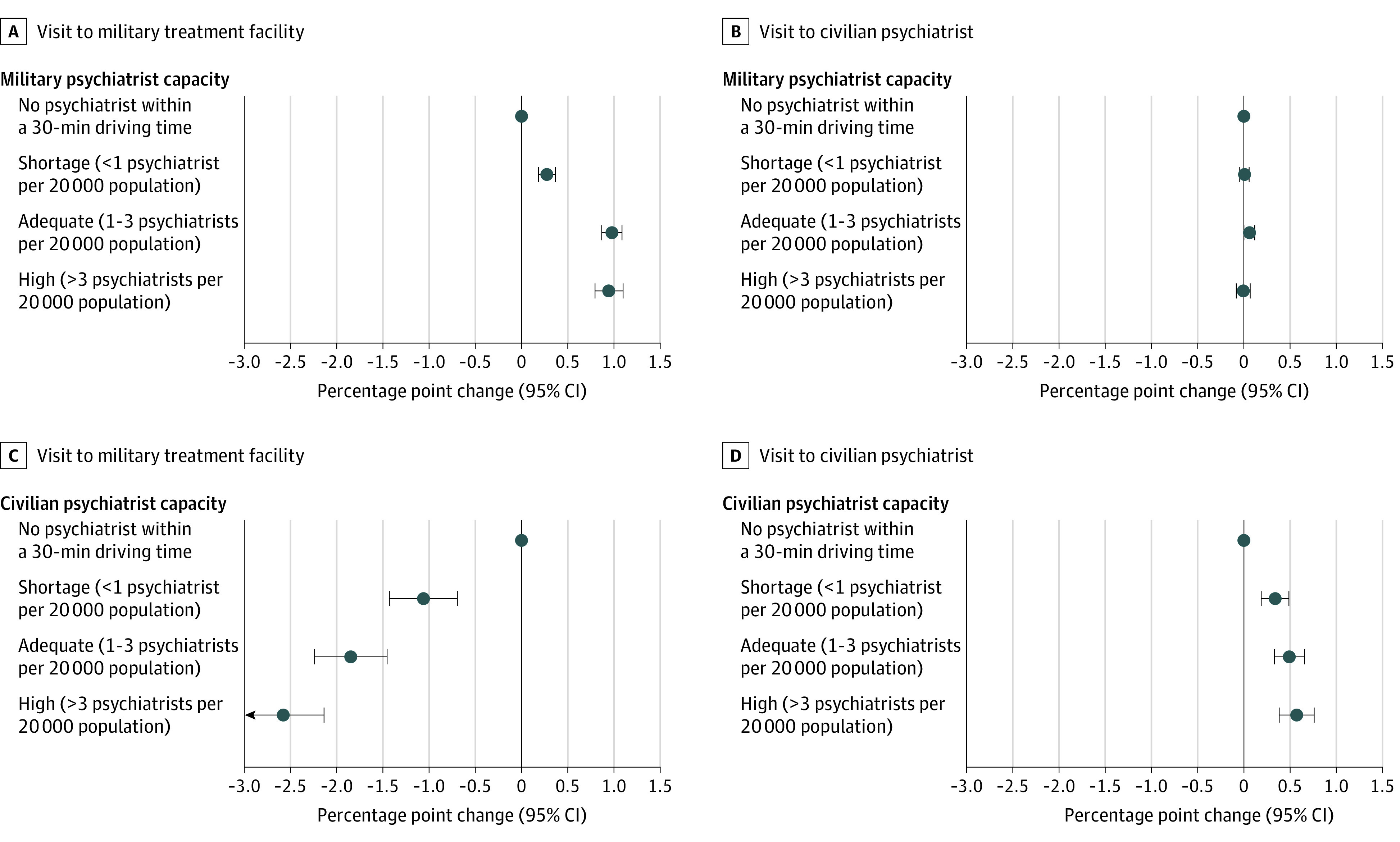
Changes in Individual Service Members’ Probability of Mental Health Care Visits by Community Psychiatrist Capacity The x-axis indicates percentage point changes relative to the reference group. The mean quarterly visit rate for the reference group is 7% to military treatment facilities and 2% to civilian psychiatrists. Error bars indicate 95% CIs.

Figure 2 shows the results from the key capacity measures from our 2-dimensional fixed-effects models (Table 2 presents the full results). The mean quarterly mental health care visit rates to military treatment facilities and civilian settings were 7% and 2%, respectively. Focusing on changes in military treatment facility psychiatric capacity, Figure 2A shows that when a service member moved from a community with zero military treatment facility psychiatrists to a community with shortages (or when the same community’s military treatment facility capacity changed from zero to shortage), the probability of a military treatment facility mental health visit increased by 0.28 percentage points (95% CI, 0.18-0.37 percentage points), which is equivalent to a 4% increase (given the 7% base rate). The change in probability of a mental health care visit to a military treatment facility increased by 0.98 percentage points (95% CI, 0.87-1.09 percentage points) when comparing adequate military treatment facility capacity communities with zero capacity (equivalent to a 14% increase) and by 0.95 percentage points (95% CI, 0.79-1.10 percentage points) when comparing high military treatment facility capacity communities with zero capacity (equivalent to a 14% increase). The probability of visits to civilian psychiatrists did not change when military treatment facility psychiatrist capacity changed (Figure 2B).

**Table 2.** Risk-Adjusted Percentage Point Changes in Probability of Mental Health Visit When Psychiatrist Capacity Changes^a^

Characteristic	Coefficient (95% CI), percentage points	
	Mental health visit made in military treatment facility	Mental health visit made to civilian psychiatrist
Mean quarterly rate at reference group, %	7	2
Military treatment facility psychiatrist capacity		
No psychiatrist within 30 min	0	0
<1 Psychiatrist per 20 000	0.28 (0.18 to 0.37)^b^	0.01 (−0.05 to 0.06)
1-3 Psychiatrists per 20 000	0.98 (0.87 to 1.09)^b^	0.06 (−0.00 to 0.12)
>3 Psychiatrists per 20 000	0.95 (0.79 to 1.10)^b^	−0.01 (−0.08 to 0.07)
Civilian psychiatrist capacity		
No psychiatrist within 30 min	0	0
<1 Psychiatrist per 20 000	−1.06 (−1.43 to −0.69)^b^	0.34 (0.19 to 0.49)^b^
1-3 Psychiatrists per 20 000	−1.85 (−2.24 to −1.45)^b^	0.49 (0.33 to 0.65)^b^
>3 Psychiatrists per 20 000	−2.58 (−3.02 to −2.14)^b^	0.57 (0.38 to 0.76)^b^
Time-varying individual events		
Married during the study period	0.38 (0.32 to 0.45)^b^	−0.05 (−0.08 to −0.02)^b^
Divorced during the quarter	0.46 (0.32 to 0.59)^b^	−0.09 (−0.17 to −0.02)^c^
Married during the quarter	−0.05 (−0.12 to 0.02)	−0.04 (−0.08 to −0.01)^c^
Demoted during the quarter	15.08 (14.81 to 15.34)^b^	1.78 (1.64 to 1.92)^b^
Gained a child during the quarter	−0.40 (−0.46 to −0.34)^b^	−0.10 (−0.13 to −0.07)^b^
Moved during the quarter	−0.72 (−0.75 to −0.69)^b^	−0.18 (−0.20 to −0.17)^b^
Return from overseas deployment in prior quarter	−0.14 (−0.24 to −0.04)^b^	−0.27 (−0.32 to −0.22)^b^
Return from overseas deployment in current quarter	−4.03 (−4.10 to −3.96)^b^	−1.05 (−1.08 to −1.02)^b^
Time trend (reference first quarter 2016)	0	0
2016		
Second quarter	0.33 (0.28 to 0.38)^b^	0.07 (0.04 to 0.09)^b^
Third quarter	0.72 (0.66 to 0.78)^b^	0.15 (0.12 to 0.18)^b^
Fourth quarter	1.16 (1.10 to 1.22)^b^	0.21 (0.18 to 0.24)^b^
2017		
First quarter	2.07 (2.01 to 2.13)^b^	0.36 (0.33 to 0.39)^b^
Second quarter	2.41 (2.35 to 2.48)^b^	0.52 (0.49 to 0.55)^b^
Third quarter	2.53 (2.46 to 2.59)^b^	0.62 (0.59 to 0.65)^b^
Fourth quarter	2.63 (2.56 to 2.69)^b^	0.71 (0.68 to 0.74)^b^
2018		
First quarter	3.37 (3.30 to 3.43)^b^	0.86 (0.83 to 0.89)^b^
Second quarter	3.79 (3.72 to 3.86)^b^	1.03 (1.00 to 1.06)^b^
Third quarter	3.98 (3.91 to 4.04)^b^	1.20 (1.16 to 1.23)^b^
Fourth quarter	4.33 (4.26 to 4.39)^b^	1.26 (1.23 to 1.29)^b^
2019		
First quarter	5.14 (5.07 to 5.21)^b^	1.46 (1.42 to 1.49)^b^
Second quarter	5.55 (5.48 to 5.62)^b^	1.68 (1.64 to 1.72)^b^
Third quarter	5.90 (5.83 to 5.97)^b^	1.93 (1.89 to 1.97)^b^
Fourth quarter	6.18 (6.11 to 6.25)^b^	2.08 (2.04 to 2.11)^b^
2020		
First quarter	6.72 (6.65 to 6.80)^b^	2.27 (2.23 to 2.31)^b^
Second quarter	5.57 (5.49 to 5.64)^b^	2.01 (1.97 to 2.05)^b^
Third quarter	6.53 (6.46 to 6.61)^b^	2.39 (2.35 to 2.43)^b^
Constant term	6.17 (5.77 to 6.57)^b^	0.27 (0.10 to 0.44)^b^
No.	22 158 647	22 158 647

^a^ Models include individual fixed effects and community fixed effects to account for unobserved differences across individuals and across communities. Robust SEs assume clustering at individual and community levels.

^b^ *P* < .01.

^c^ *P* < .05.

The probability of a visit to a military treatment facility decreased as civilian psychiatrist capacity increased (Figure 2C). When a service member moved from a community with zero civilian capacity to a community with a civilian shortage, the probability of a military treatment facility visit decreased by 1.06 percentage points (95% CI, −1.43 to −0.69 percentage points), equivalent to a 15% decrease, when holding constant their military psychiatric capacity. When the service member moved from a community with zero civilian capacity to a high civilian capacity community, the probability of a military treatment facility visit decreased by 2.58 percentage points (95% CI, −3.02 to −2.14 percentage points), equivalent to a 35% decrease. Compared with zero civilian capacity, the probability of a civilian psychiatrist visit is 0.34 percentage points (95% CI, 0.19-0.49 percentage points) higher in shortage areas, equivalent to a 19% increase, and 0.57 percentage points (95% CI, 0.38-0.76 percentage points) higher in high civilian capacity areas, equivalent to a 32% increase (Figure 2D). The difference in the effect sizes of military treatment facility and civilian capacities suggested that military and civilian psychiatrists are not perfect substitutes.

**Figure 3.**
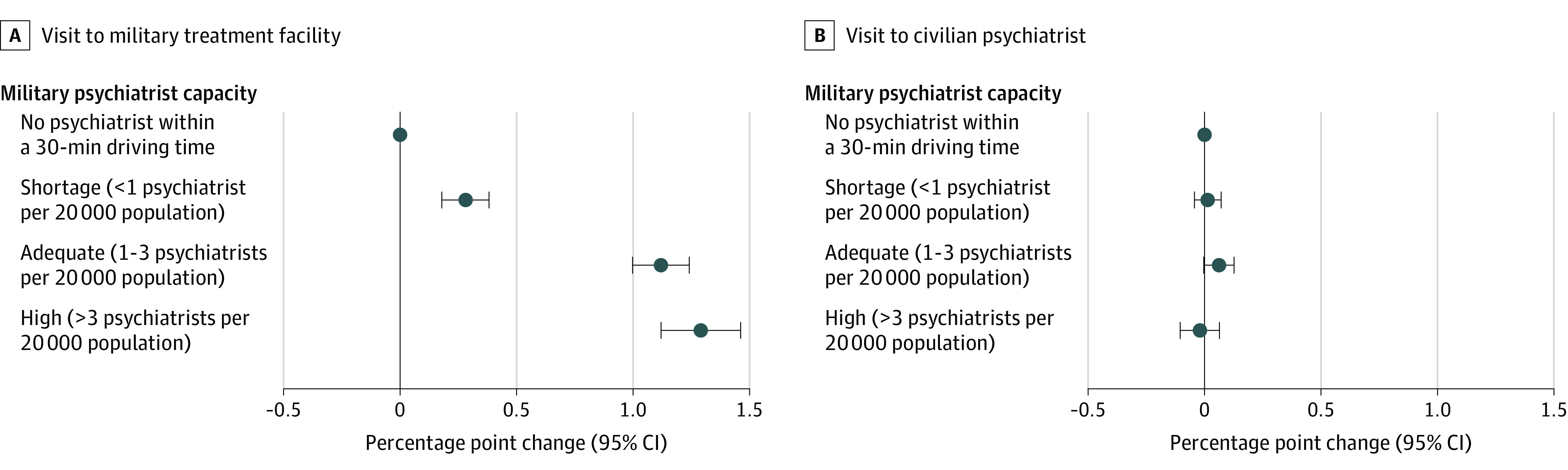
Changes in Individual Service Members’ Probability of Mental Health Care Visits by Military Treatment Facility Psychiatrist Capacity When Civilian Capacity Is High The x-axis indicates percentage point changes relative to the reference group. The mean quarterly visit rate for the reference group is 6% to military treatment facilities and 3% to civilian psychiatrists. Error bars indicate 95% CIs.

Next, we investigated whether the association between military treatment facility visit rate and military treatment facility capacity level in Figure 2 would diminish when limiting the analysis to communities with adequate or high civilian capacity because it would be easier to substitute military care with civilian care in such communities. Figure 3 (full results in eTable 1 in Supplement 1) shows that the same pattern continues to hold. The mean (SD) quarterly rate of mental health care visits to military treatment facilities was similar between this restricted analysis and the full analysis (6% vs 7%). Despite living in communities with easy geographical access to civilian psychiatrists, service members in communities with high military treatment facility capacity increased their military treatment facility visit rate by 1.29 percentage points (95% CI, 1.12-1.55 percentage points), equivalent to a 21% increase, compared with those with zero military treatment facility psychiatrists.

Our measure of civilian psychiatrist supply may overestimate private sector capacity because only 37% of civilian behavioral health professionals accepted new TRICARE patients.^16,17^ We reestimated our model assuming that private capacity was 37% and found similar results, except the decrease in military treatment facility visits associated with civilian capacity increases was smaller and closer in magnitude to the effect size of military treatment facility capacity (eTable 2 in Supplement 1). Our third sensitivity analysis limited the sample to only service members who moved at least once during the study period, so we could focus on variations in capacity that were associated with movers changing communities. Our results remained robust (eTable 3 in Supplement 1). We provide results from additional sensitivity analysis in eAppendix 2, eTable 4, and eTable 5 in Supplement 1.

## Discussion

Our analysis of 1 958 421 service members who were followed up between January 1, 2016, and September 30, 2020, showed that 13% of our study population lived in communities with no military treatment facility psychiatrists within a 30-minute driving time and that 5% lived in communities with no civilian psychiatrist capacity within a 30-minute driving time. Approximately three-fourths of all mental health care visits occurred in military treatment facilities. When military treatment facility psychiatrist capacity changed from zero to adequate or high capacity, the probability of visits to military treatment facilities increased by 14%, while the probability of visits to civilian psychiatrists did not change. When civilian psychiatrist capacity changed from zero to adequate or high capacity, the probability of mental health visits to military treatment facilities decreased by 26% to 35%, and the probability of visits to civilian psychiatrists increased by 27% to 32%.

Our results have several implications. First, with three-fourths of mental health care visits occurring in military treatment facilities, it would be difficult for TRICARE to rely on the civilian sector to fulfill the mental health needs of active duty service members. Even in high civilian capacity communities, more than two-thirds of mental health care visits by the active duty population occurred in military treatment facilities. This could be due to other barriers of access to civilian psychiatrists, such as coordination of care and low acceptance rates of TRICARE patients.^17^ Second, if the goal is to encourage more mental health care use by service members, it would appear that increasing psychiatrist capacity within the military health system would be the first-order solution. However, a recent study projected a continuing shortage of psychiatric beds^30^ and of the psychiatrist workforce into 2030, but the Health Resources & Service Administration projected an increase in the number of nurse practitioners.^31^ Future research should identify strategies to use nurse practitioners to improve the mental health care needs of the military population.

Our findings echoed previous work that suggested policymakers and the Defense Health Agency could increase efficiency by focusing on improving specialty services capacity on the military side (in our case, psychiatrists).^18^ Our results show that responses are similar once the threshold for adequate capacity is passed. With 13% and 66% of active duty service members living in communities with zero or a shortage of military treatment facility psychiatrists within a 30-minute driving time, respectively, one potential strategy might be realigning some military treatment facility psychiatrists in areas with high capacity from the same state to support beneficiaries in communities with shortages via telemedicine. Such an approach would align with the National Defense Authorization Act of 2017 requirement to enhance telehealth in the military health system.^32^ Understanding the growth and efficacy of this delivery modality in the military health system in the post–COVID-19 pandemic era is critical, but it is beyond the scope of this article. Other alternatives might include the establishment of mobile clinics or embedding psychiatrist specialists in primary care clinics, which has found success in the Department of Veterans Affairs system in treating veterans with psychiatric illness.^33^ These initiatives might yield different results depending on the region.

Given that more than two-thirds of the military population are in communities with high civilian psychiatrist capacity, the civilian health care sector remains an important partner in the military health care delivery system. We found that the probability of mental health care visits to the civilian setting did increase when civilian psychiatric capacity increased. However, because visits to civilian psychiatrists only represent a small fraction of overall care visits, the absolute magnitude of change is small. Taken together, our findings suggest that policies that address nongeographical barriers in the civilian sector remain critical to achieve the optimal balance between military and civilian care provision. For instance, higher reimbursement rates to civilian psychiatrists could improve service members’ ability to find psychiatrists that accept TRICARE. Health care professionals outside the TRICARE network are generally reimbursed on a fee schedule similar to Medicare, while civilian in-network professionals receive a lower reimbursement.^5^ Health care professional surveys consistently show that less than 40% of behavioral health professionals (including psychiatrists) accepted new TRICARE patients^16,17^ compared with 60% and 59% of psychiatrists accepting new Medicare and private insurance patients, respectively, in recent years.^34^ Other policy examples might include better coordination of care, electronic sharing of deployment and case history, and reduced waiting time for referrals and during visits. These policies would encourage beneficiaries to use available civilian capacity, especially in communities that lack military treatment facility access but have robust civilian capacity.

### Limitations

This study has some limitations. First, we recognize that there is a broader set of health care professionals for mental health care. Primary care physicians, physician assistants, and nurse practitioners can diagnose and prescribe medications to treat mental health conditions; professionals such as clinical psychologists, social workers, and therapists provide nonmedical treatments. We focus on psychiatrists because there are well-defined thresholds for shortage and high capacity for psychiatrists that can be applied consistently to both military and civilian capacity measures. Care-seeking responses to this broader set of health care professional capacity merits a separate analysis. Furthermore, focusing on psychiatrist capacity reduces estimation error, given that civilian physician data are more reliable than nonphysician data.

Second, we might not have captured 100% of medical visits in the civilian sector if the service member opted to see a civilian health care professional without using TRICARE. For example, service members may have dual coverage (eg, insurance from their spouse’s employer) to obtain civilian care outside of TRICARE. This might explain why the reduction in probability of a visit to a military treatment facility was not completely offset by a comparable increase in visits to civilian facilities when civilian capacity increased. It remains unclear how often service members pay out of pocket or have dual coverage. We are also not aware of studies documenting systematic differences in underreporting across communities. Our models partially address this limitation by including individual fixed effects, which would control for personal preferences for such care choices but would not completely eliminate this bias.

Third, we had limited data to capture stressful events and could not capture events such as recent deployment to a hostile environment or financial hardship. We also did not have measures of other types of access barriers (eg, local measures of civilian psychiatrists’ TRICARE acceptance rates) to explore mechanisms behind the association we observed. Our results should be interpreted as measuring the mean change in mental health care visit rates when local psychiatrist capacity changed.

Fourth, our outcome measured actual visits to psychiatrists, which might not reflect true mental health care needs. Stigma associated with seeking care can result in an inverse association between perceived and actual need for care.^35,36^ Individual fixed effects control for time-invariant perceived need for care among service members but not when this perceived need changes. Our model coefficients likely underestimated the associations between capacity and the true mental health needs of the US military.

Fifth, our capacity measure was based on a 30-minute driving time, which might become invalid after March 2020 due to the increase in telehealth. Although our results remained robust when we eliminated observations on and after March 2020, it would be important to investigate the role of telehealth in the post–COVID-19 pandemic era.

Sixth, our study population included only active duty service members, which represents only 15% of TRICARE beneficiaries. Our results cannot be generalized to military retirees or other non–active duty beneficiaries. Individual characteristics and underlying mental health needs are quite different between active duty and non–active duty beneficiaries and warrant a separate analysis.

## Conclusions

In this cohort study of 1 958 421 active duty service members who were followed up between January 2016 and September 2020, we found that mental health care use in military treatment facilities and civilian settings changed significantly when military treatment facility and civilian psychiatrist capacity changed. Service members largely rely on military treatment facilities for their mental health care needs; in any given quarter, approximately three-fourths of all mental health care visits occurred in military treatment facilities. Although more than two-thirds of service members live in communities of high civilian capacity, use of civilian care remains low and the association between the visit rate to military treatment facilities and military treatment facility capacity changes remains strong in these communities. Our findings suggest that realigning military treatment facility psychiatrists across communities with shortages and high capacity military treatment facilities would be beneficial. Furthermore, it is equally important to address nongeographical barriers to accessing civilian psychiatrists, particularly in communities that lack military treatment facility access but have robust civilian capacity.

## Notes

Supplement 1. **eAppendix 1.** Data and Methods to Define Relevant Geographic Access Area From Patient Perspective **eAppendix 2.** Additional Sensitivity Analysis
eReferences.
**eTable 1.** Risk-Adjusted Percentage Point Changes in Probability of Mental Health Visit When Psychiatrist Capacity Changes, Restricted to Communities With High Civilian Capacity **eTable 2.** Risk-Adjusted Percentage Point Changes in Probability of Mental Health Visit When Psychiatrist Capacity Changes: Assuming Only 37% Civilian Psychiatrists Accept TRICARE Patients **eTable 3.** Risk-Adjusted Percentage Point Changes in Probability of Mental Health Visit When Psychiatrist Capacity Changes: Restricted to Service Members Who Had Moved at Least Once **eTable 4.** Risk-Adjusted Percentage Point Changes in Probability of Mental Health Visit When Psychiatrist Capacity Changes: Restricted Mental Health Diagnoses **eTable 5.** Risk-Adjusted Percentage Point Changes in Probability of Mental Health Visit When Psychiatrist Capacity Changes: Separate Estimate for Increased and Decreased Capacity

Supplement 2.
Data Sharing Statement
