# Supplementary material for: Propensity of US Military Personnel to Seek Mental Health Care When Community Psychiatric Capacity Changes
Source: JAMA Health Forum. 2023 Oct 6;4(10):e233330. doi: 10.1001/jamahealthforum.2023.3330 (PMC10559180; doi:10.1001/jamahealthforum.2023.3330)
Supplement: Supplement 1. — eAppendix 1. Data and Methods to Define Relevant Geographic Access Area From Patient Perspective eAppendix 2. Additional Sensitivity Analysis eReferences. eTable 1. Risk-Adjusted Percentage Point Changes in Probability of Mental Health Visit When Psychiatrist Capacity Changes, Restricted to Communities With High Civilian Capacity eTable 2. Risk-Adjusted Percentage Point Changes in Probability of Mental Health Visit When Psychiatrist Capacity Changes: Assuming Only 37% Civilian Psychiatrists Accept TRICARE Patients eTable 3. Risk-Adjusted Percentage Point Changes in Probability of Mental Health Visit When Psychiatrist Capacity Changes: Restricted to Service Members Who Had Moved at Least Once eTable 4. Risk-Adjusted Percentage Point Changes in Probability of Mental Health Visit When Psychiatrist Capacity Changes: Restricted Mental Health Diagnoses eTable 5. Risk-Adjusted Percentage Point Changes in Probability of Mental Health Visit When Psychiatrist Capacity Changes: Separate Estimate for Increased and Decreased Capacity [file jamahealthforum-e233330-s001.pdf]

Supplementary Online Content

Shen YC, Bacolod M, Heissel JA. Propensity of US military personnel to seek mental health care when community psychiatric capacity changes. *JAMA Health Forum.* 2023;4(10):e233330. doi:10.1001/jamahealthforum.2023.3330

eAppendix 1. Data and Methods to Define Relevant Geographic Access Area From Patient Perspective
eAppendix 2. Additional Sensitivity Analysis
eReferences.
eTable 1. Risk-Adjusted Percentage Point Changes in Probability of Mental Health Visit When Psychiatrist Capacity Changes, Restricted to Communities With High Civilian Capacity
eTable 2. Risk-Adjusted Percentage Point Changes in Probability of Mental Health Visit When Psychiatrist Capacity Changes: Assuming Only 37% Civilian Psychiatrists Accept TRICARE Patients
eTable 3. Risk-Adjusted Percentage Point Changes in Probability of Mental Health Visit When Psychiatrist Capacity Changes: Restricted to Service Members Who Had Moved at Least Once
eTable 4. Risk-Adjusted Percentage Point Changes in Probability of Mental Health Visit When Psychiatrist Capacity Changes: Restricted Mental Health Diagnoses
eTable 5. Risk-Adjusted Percentage Point Changes in Probability of Mental Health Visit When Psychiatrist Capacity Changes: Separate Estimate for Increased and Decreased Capacity

This supplementary material has been provided by the authors to give readers additional information about their work.

© 2023 Shen YC et al. *JAMA Health Forum.*

eAppendix 1. Data and Methods to Define Relevant Geographic Access Area From Patient Perspective

We combine monthly data from January 2016 to September 2020 to form our analysis. First, we use the Defense Enrollment Eligibility Reporting System (DEERS) to capture beneficiary population size, demographic, and military branch of service characteristics of TRICARE beneficiaries for a given community. Second, we use the Medical Expense and Performance Reporting System (MEPRS) and the Defense Medical Human Resource System internet (DMHRSi) to capture MTF psychiatrist capacity; and the National Plan and Provider Enumeration System National Provider Identifier (NPI) data to identify civilian psychiatrists and their practice ZIP code. Third, we use the U.S. Census, American Community Survey (ACS), and the Social Determinants of Health Database to capture the community's overall population and socioeconomic characteristics.[1] Finally, we use a web-based query[2] to derive database of driving time between centers of each ZIP code community and (1) the MTF's and (2) ZIP code centers of civilian psychiatrists' practicing location.

We use ZIP codes to define community because it is the smallest geographic unit we can capture across data sources. However, the closest psychiatrist is not necessarily practicing in the same ZIP code as the patient, especially in cities, so we need to define a more robust geographic boundary to assess patients' access to psychiatrists. Rather than using the catchment area approach which defines relevant geographic boundaries from a facility's perspective, we define geographic coverage from the patient's perspective. For each community, we use travel time concept to define the relevant access area, rather than alternative definitions such as political boundaries (i.e., state or county lines) or fixed radius approach (such as the 20-mile radius catchment area used by the Defense Health Agency) for several reasons. First, using travel time does not impose arbitrary limits on actual access like political boundaries—an Idaho patient who

© 2023 Shen YC et al. *JAMA Health Forum.*

lives near the state line of Oregon would have easier access to a psychiatrist in a certain part of Oregon than in Idaho. Second, unlike a fixed-mile radius approach which cannot capture geographical barriers such as mountains and water bodies, the driving time approach captures geographic access more accurately and consistently. Third, travel time is positively associated with treatment attrition. For example, female veterans are more likely to attrite from Veteran's Heath Administration care the longer their drive time, particularly for new patients[3], and longer travel time is associated with fewer annual visits for depression treatment.[4]

Researchers have used 30 minutes of travel time as a benchmark for various medical treatments such as emergency caesarean delivery,[5] cardiac care,[6,7] and opioid treatment[8]; while others have used a linear measure of time and its relationship to care.[3,4,9–11] The U.S. Department of Health and Human Services uses 30 minutes travel time as the rational area of coverage for primary care and 40 minutes for dental and mental health care.[12] We chose 30 minutes car driving time (1-hour round trip) as a reasonable time threshold that TRICARE beneficiaries are willing to travel on a repeated basis since each mental health episode can require multiple follow-up visits. It should be noted that while driving time accounts for large percent of travel time, actual travel time would be longer than the 30-minute driving time due to various factors, such as wait time for the ride, navigating parking structure, and in the case of public transportation, frequent stops. In our sensitivity analysis, we use 40 minutes of driving time as alternative threshold.

Based on the above geographic access definition, we take the following steps to capture the collection of ZIP codes and MTFs with psychiatrists that are within a 30-minute driving time of a given community. First, for each ZIP code where we have a TRICARE beneficiary or civilian providers, we obtain longitude and latitude coordinates of the ZIP code interior center

© 2023 Shen YC et al. *JAMA Health Forum.*

based on the US Census. Second, for each MTF, we obtain longitude and latitude coordinates via automated web interface based on their physical address. Last, we derived a travel-time database using web-based queries that identify driving time under normal traffic conditions between each pair (ZIP code to ZIP code for care through a civilian provider and ZIP code to MTF for MTF-provided care). All psychiatrists practicing in ZIP codes and MTFs that can be reached within a 30-minute driving time from a given community would be considered geographically accessible to beneficiaries for that community.

Based on our geographic access definition, we derived a drive-time database using web-based queries that identify driving time under normal traffic conditions between each pair based on their longitude and latitude coordinates (ZIP code to ZIP code for care through a civilian provider and ZIP code to MTF for MTF-provided care).[2] All psychiatrists practicing in ZIP codes and MTFs that can be reached within a 30-minute driving time from a given community would be considered geographically accessible to beneficiaries for that community.

© 2023 Shen YC et al. *JAMA Health Forum.*

eAppendix 2. Additional Sensitivity Analysis

We recognize that psychiatrists are not the only providers for treating mental health illnesses, and many mental health conditions included in our outcome measure can be treated by a combination of psychiatrists and/or other providers, such as clinical psychologists, psychiatric nurse practitioners, etc. However, there is no clearly defined subset of conditions in which psychiatrists are the dominant providers. As an illustrative example we limit the mental health diagnoses to only the following: bipolar disorder, PTSD, major depression, drug or alcohol misuse, and psychotic disorders. Appendix Table 4 shows that the base rate and the absolute change in percentage point all go down proportionally, so the relative change remains the same—when a service member moves from a zero MTF psychiatrist community to one with adequate number, probability of visit goes up by 15% (0.4 percentage point increase off the base of 2.68%).

Lastly, in our main model, we estimate the average effect of capacity change, without differentiating the direction of the change (i.e., we assume symmetrical response). We investigate whether the effect is symmetric by estimating a separate set of coefficients for when the change is in the decreasing direction. Appendix Table 5 shows that for MTF capacity, the response is indeed asymmetric—response is bigger when capacity increased and smaller when capacity decreased; for civilian capacity, the response is similar whether capacity increased or decreased.

© 2023 Shen YC et al. *JAMA Health Forum.*

eReferences.

1. Agency for Healthcare Research and Quality. Social Determinants of Health Database (Beta Version). Published June 2021. Accessed June 29, 2021. http://www.ahrq.gov/sdoh/data-analytics/sdoh-data.html

2. Developer Guide - HERE Routing API. HERE Developer. Accessed April 30, 2022. https://developer.here.com/documentation/routing-api/dev_guide/index.html

3. Friedman SA, Frayne SM, Berg E, et al. Travel time and attrition from VHA care among women Veterans: How far is too far? *Med Care*. 2015;53(4 0 1):S15-S22. doi:10.1097/MLR.0000000000000296

4. Fortney J, Rost K, Zhang M, Warren J. The Impact of Geographic Accessibility on the Intensity and Quality of Depression Treatment. *Medical Care*. 1999;37(9):884-893.

5. Uribe-Leitz T, Matsas B, Dalton MK, et al. Geospatial Analysis of Access to Emergency Cesarean Delivery for Military and Civilian Populations in the US. *JAMA Network Open*. 2022;5(1):e2142835. doi:10.1001/jamanetworkopen.2021.42835

6. Sommerhalter KM, Insaf TZ, Akkaya-Hocagil T, et al. Proximity to Pediatric Cardiac Surgical Care among Adolescents with Congenital Heart Defects in 11 New York Counties. *Birth Defects Research*. 2017;109(18):1494-1503. doi:10.1002/bdr2.1129

7. Graves BA. Geographic Analysis of Cardiac Interventional Services in Alabama. *Journal of Cardiovascular Nursing*. 2011;26(4):E1. doi:10.1097/JCN.0b013e3181ecaacb

8. Hyder A, Lee J, Dundon A, et al. Opioid Treatment Deserts: Concept development and application in a US Midwestern urban county. *PLOS ONE*. 2021;16(5):e0250324. doi:10.1371/journal.pone.0250324

9. Chan L, Hart LG, Goodman DC. Geographic Access to Health Care for Rural Medicare Beneficiaries. *The Journal of Rural Health*. 2006;22(2):140-146. doi:10.1111/j.1748-0361.2006.00022.x

10. Lee CS, Morris A, Van Gelder RN, Lee AY. Evaluating Access to Eye Care in the Contiguous United States by Calculated Driving Time in the United States Medicare Population. *Ophthalmology*. 2016;123(12):2456-2461. doi:10.1016/j.ophtha.2016.08.015

11. Kleinman RA. Comparison of Driving Times to Opioid Treatment Programs and Pharmacies in the US. *JAMA Psychiatry*. 2020;77(11):1163-1171. doi:10.1001/jamapsychiatry.2020.1624

12. *42 CFR Part 5 -- Designation of Health Professional(s) Shortage Areas*. Vol 45 FR 76000.; 1980. Accessed April 28, 2022. https://www.ecfr.gov/current/title-42/chapter-I/subchapter-A/part-5

© 2023 Shen YC et al. *JAMA Health Forum.*

eTable 1. Risk-Adjusted Percentage Point Changes in Probability of Mental Health Visit When Psychiatrist Capacity Changes, Restricted to Communities With High Civilian Capacity

	**Mental health visit made in MTF**	**Mental health visit made in civilian**
Mean quarterly rate at reference group	6.21%	3.05%
**MTF psychiatrist capacity**		
no psychiatrist within 30-min	0.00	0.00
<1 psychiatrist per 20K	0.28**	0.01
	[0.18,0.38]	[-0.04,0.07]
1-3 psychiatrist per 20K	1.12**	0.06
	[1.00,1.24]	[-0.00,0.13]
>3 psychiatrist per 20K	1.29**	-0.02
	[1.12,1.46]	[-0.10,0.06]
**Time-varying individual events**		
Married during the study period	0.30**	-0.06**
	[0.23,0.38]	[-0.09,-0.02]
Got divorced during the quarter	0.43**	-0.09*
	[0.29,0.58]	[-0.17,-0.01]
Got married during the quarter	-0.10*	-0.06**
	[-0.18,-0.02]	[-0.11,-0.02]
Demoted during the quarter	14.17**	1.74**
	[13.87,14.46]	[1.59,1.90]
Gained a child during the quarter	-0.42**	-0.10**
	[-0.48,-0.35]	[-0.14,-0.07]
Moved during the quarter	-0.75**	-0.18**
	[-0.79,-0.71]	[-0.20,-0.16]
Return from oversea deployment in prior quarter	-0.15**	-0.27**
	[-0.26,-0.04]	[-0.32,-0.22]
Return from oversea deployment in current quarter	-3.90**	-1.03**
	[-3.98,-3.82]	[-1.07,-1.00]
Time trend (reference is 2016 Q1)	0.00	0.00
2016 Q2	0.33**	0.08**
	[0.28,0.39]	[0.05,0.11]
2016 Q3	0.70**	0.18**
	[0.64,0.76]	[0.15,0.20]
2016 Q4	1.11**	0.25**

	[1.05,1.17]	[0.22,0.28]
2017 Q1	2.00**	0.40**
	[1.94,2.07]	[0.37,0.43]
2017 Q2	2.32**	0.53**
	[2.25,2.38]	[0.50,0.56]
2017 Q3	2.43**	0.64**
	[2.37,2.50]	[0.61,0.67]
2017 Q4	2.56**	0.72**
	[2.49,2.63]	[0.69,0.76]
2018 Q1	3.25**	0.87**
	[3.18,3.33]	[0.84,0.91]
2018 Q2	3.65**	1.03**
	[3.58,3.72]	[1.00,1.07]
2018 Q3	3.86**	1.21**
	[3.79,3.94]	[1.18,1.25]
2018 Q4	4.23**	1.28**
	[4.16,4.30]	[1.25,1.32]
2019 Q1	5.08**	1.49**
	[5.00,5.15]	[1.45,1.53]
2019 Q2	5.44**	1.72**
	[5.37,5.52]	[1.68,1.76]
2019 Q3	5.80**	1.96**
	[5.73,5.88]	[1.92,2.01]
2019 Q4	6.10**	2.13**
	[6.02,6.17]	[2.09,2.17]
2020 Q1	6.63**	2.35**
	[6.55,6.71]	[2.31,2.39]
2020 Q2	5.42**	2.09**
	[5.34,5.50]	[2.05,2.13]
2020 Q3	6.38**	2.48**
	[6.30,6.46]	[2.44,2.53]
Constant term	3.97**	0.70**
	[3.86,4.08]	[0.64,0.76]
N	18,638,427	18,638,427

Note: * p<0.05 ** p<0.01. Models include individual fixed effects and community fixed effects to account for unobserved differences across individuals and across communities. Robust standard errors assume clustering at individual and community levels.

© 2023 Shen YC et al. *JAMA Health Forum.*

eTable 2. Risk-Adjusted Percentage Point Changes in Probability of Mental Health Visit When Psychiatrist Capacity Changes: Assuming Only 37% Civilian Psychiatrists Accept TRICARE Patients

	Mental health visit made in MTF	Mental health visit made in civilian
Mean quarterly rate at reference group	7.1%	1.8%
**MTF psychiatrist capacity**		
no psychiatrist within 30-min	0.00	0.00
<1 psychiatrist per 20K	0.26**	0.01
	[0.17,0.35]	[-0.04,0.06]
1-3 psychiatrist per 20K	0.96**	0.06
	[0.85,1.07]	[-0.00,0.11]
>3 psychiatrist per 20K	0.93**	-0.01
	[0.78,1.08]	[-0.08,0.07]
**Civilian psychiatrist capacity**		
no psychiatrist within 30-min	0.00	0.00
<1 psychiatrist per 20K	-1.09**	0.34**
	[-1.45,-0.72]	[0.19,0.49]
1-3 psychiatrist per 20K	-0.69**	0.43**
	[-1.12,-0.27]	[0.23,0.62]
>3 psychiatrist per 20K	-0.52*	0.54**
	[-0.98,-0.07]	[0.33,0.75]
N	22,158,647	22,158,647

Note: * p<0.05 ** p<0.01. Model specifications are identical as Table 2. Robust standard errors assume clustering at individual and community levels.

eTable 3. Risk-Adjusted Percentage Point Changes in Probability of Mental Health Visit When Psychiatrist Capacity Changes: Restricted to Service Members Who Had Moved at Least Once

	**Mental health visit made in MTF**	**Mental health visit made in civilian**
Mean quarterly rate at reference group	6.66%	1.64%
**MTF psychiatrist capacity**		
no psychiatrist within 30-min	0.00	0.00
<1 psychiatrist per 20K	0.35**	0.04
	[0.25,0.45]	[-0.02,0.09]
1-3 psychiatrist per 20K	0.94**	0.08**
	[0.82,1.06]	[0.02,0.15]
>3 psychiatrist per 20K	0.85**	0.04
	[0.68,1.01]	[-0.04,0.13]
**Civilian psychiatrist capacity**		
no psychiatrist within 30-min	0.00	0.00
<1 psychiatrist per 20K	-0.93**	0.31**
	[-1.32,-0.55]	[0.15,0.47]
1-3 psychiatrist per 20K	-1.42**	0.54**
	[-1.83,-1.01]	[0.36,0.71]
>3 psychiatrist per 20K	-2.30**	0.52**
	[-2.77,-1.82]	[0.31,0.72]
**Time-varying individual events**		
Married during the study period	0.42**	-0.04
	[0.34,0.49]	[-0.07,0.00]
Got divorced during the quarter	0.50**	-0.09*
	[0.34,0.65]	[-0.18,-0.00]
Got married during the quarter	-0.06	-0.04*
	[-0.14,0.02]	[-0.08,-0.00]
Demoted during the quarter	15.13**	1.77**
	[14.82,15.45]	[1.61,1.94]
Gained a child during the quarter	-0.39**	-0.11**
	[-0.46,-0.33]	[-0.14,-0.07]
Moved during the quarter	-0.72**	-0.19**
	[-0.75,-0.68]	[-0.20,-0.17]
Return from oversea deployment in prior quarter	0.07	-0.25**
	[-0.05,0.19]	[-0.31,-0.19]
Return from oversea deployment in current quarter	-3.85**	-1.01**

© 2023 Shen YC et al. *JAMA Health Forum.*

	[-3.93,-3.77]	[-1.05,-0.97]
Time trend (reference is 2016 Q1)	0.00	0.00
2016 Q2	0.42**	0.09**
	[0.36,0.48]	[0.06,0.12]
2016 Q3	0.79**	0.17**
	[0.73,0.85]	[0.14,0.20]
2016 Q4	1.11**	0.21**
	[1.04,1.18]	[0.18,0.24]
2017 Q1	1.90**	0.35**
	[1.83,1.97]	[0.32,0.38]
2017 Q2	2.13**	0.46**
	[2.06,2.20]	[0.43,0.49]
2017 Q3	2.30**	0.56**
	[2.23,2.37]	[0.53,0.60]
2017 Q4	2.41**	0.65**
	[2.34,2.49]	[0.62,0.69]
2018 Q1	3.06**	0.79**
	[2.99,3.14]	[0.76,0.83]
2018 Q2	3.54**	0.95**
	[3.46,3.61]	[0.92,0.99]
2018 Q3	3.83**	1.13**
	[3.75,3.90]	[1.10,1.17]
2018 Q4	4.19**	1.20**
	[4.11,4.26]	[1.16,1.24]
2019 Q1	4.97**	1.40**
	[4.89,5.05]	[1.36,1.44]
2019 Q2	5.43**	1.61**
	[5.35,5.51]	[1.57,1.65]
2019 Q3	5.82**	1.88**
	[5.74,5.90]	[1.83,1.92]
2019 Q4	6.13**	2.02**
	[6.05,6.21]	[1.98,2.06]
2020 Q1	6.66**	2.22**
	[6.58,6.74]	[2.17,2.26]
2020 Q2	5.57**	1.95**
	[5.49,5.65]	[1.91,2.00]
2020 Q3	6.54**	2.34**
	[6.46,6.62]	[2.29,2.38]
Constant term	4.90**	0.10
	[4.48,5.33]	[-0.09,0.29]

© 2023 Shen YC et al. *JAMA Health Forum.*

N	17,002,007	17,002,007

Note: * p<0.05 ** p<0.01. Models include individual fixed effects and community fixed effects to account for unobserved differences across individuals and across communities. Robust standard errors assume clustering at individual and community levels

© 2023 Shen YC et al. *JAMA Health Forum.*

eTable 4. Risk-Adjusted Percentage Point Changes in Probability of Mental Health Visit When Psychiatrist Capacity Changes: Restricted Mental Health Diagnoses

	**Selected mental health visit made in MTF**	**Selected mental health visit made in civilian**
Mean quarterly rate at reference group	2.68%	0.83%
no psychiatrist within 30-min	0.00	0.00
<1 psychiatrist per 20K	0.15**	-0.01
	[0.09,0.21]	[-0.05,0.02]
1-3 psychiatrist per 20K	0.40**	0.02
	[0.33,0.47]	[-0.02,0.06]
>3 psychiatrist per 20K	0.43**	-0.03
	[0.33,0.53]	[-0.08,0.02]
**Civilian psychiatrist capacity**		
no psychiatrist within 30-min	0.00	0.00
<1 psychiatrist per 20K	-0.32*	0.23**
	[-0.58,-0.06]	[0.12,0.33]
1-3 psychiatrist per 20K	-1.23**	0.31**
	[-1.51,-0.96]	[0.20,0.42]
>3 psychiatrist per 20K	-1.26**	0.37**
	[-1.57,-0.96]	[0.24,0.50]
**Time-varying individual events**		
Married during the study period	-0.06**	-0.08**
	[-0.11,-0.02]	[-0.11,-0.06]
Got divorced during the quarter	-0.01	-0.11**
	[-0.10,0.08]	[-0.17,-0.06]
Got married during the quarter	-0.14**	-0.07**
	[-0.19,-0.10]	[-0.09,-0.04]
Demoted during the quarter	12.66**	1.61**
	[12.42,12.90]	[1.49,1.72]
Gained a child during the quarter	-0.19**	-0.08**
	[-0.23,-0.15]	[-0.10,-0.06]
Moved during the quarter	-0.43**	-0.08**
	[-0.45,-0.41]	[-0.09,-0.06]
Return from oversea deployment in prior quarter	-0.16**	-0.09**
	[-0.22,-0.10]	[-0.12,-0.05]

© 2023 Shen YC et al. *JAMA Health Forum.*

Return from oversea deployment in current quarter	-1.63**	-0.48**
	[-1.67,-1.58]	[-0.50,-0.46]
Time trend (reference is 2016 Q1)	0.00	0.00
2016 Q2	0.19**	0.04**
	[0.16,0.22]	[0.02,0.05]
2016 Q3	0.46**	0.09**
	[0.42,0.49]	[0.07,0.11]
2016 Q4	0.80**	0.11**
	[0.77,0.84]	[0.09,0.13]
2017 Q1	1.18**	0.21**
	[1.14,1.22]	[0.19,0.23]
2017 Q2	1.47**	0.31**
	[1.43,1.51]	[0.29,0.33]
2017 Q3	1.64**	0.37**
	[1.60,1.68]	[0.35,0.39]
2017 Q4	1.72**	0.40**
	[1.68,1.76]	[0.37,0.42]
2018 Q1	2.01**	0.50**
	[1.97,2.05]	[0.48,0.52]
2018 Q2	2.17**	0.59**
	[2.13,2.21]	[0.56,0.61]
2018 Q3	2.31**	0.68**
	[2.26,2.35]	[0.66,0.70]
2018 Q4	2.50**	0.72**
	[2.45,2.54]	[0.70,0.75]
2019 Q1	2.83**	0.82**
	[2.79,2.88]	[0.80,0.85]
2019 Q2	3.07**	0.95**
	[3.03,3.12]	[0.92,0.97]
2019 Q3	3.25**	1.08**
	[3.21,3.30]	[1.05,1.10]
2019 Q4	3.38**	1.16**
	[3.33,3.42]	[1.13,1.18]
2020 Q1	3.65**	1.27**
	[3.61,3.70]	[1.24,1.30]
2020 Q2	3.33**	1.20**
	[3.28,3.38]	[1.17,1.22]
2020 Q3	3.65**	1.39**
	[3.60,3.70]	[1.36,1.42]

© 2023 Shen YC et al. *JAMA Health Forum.*

Constant term	2.07**	-0.03
	[1.79,2.35]	[-0.15,0.09]
N	22,158,647	22,158,647

Note: * p<0.05 ** p<0.01. Selected mental health diagnoses include bipolar disorder, PTSD, major depression, drug or alcohol misuse, and psychotic disorders. Models include individual fixed effects and community fixed effects to account for unobserved differences across individuals and across communities. Robust standard errors assume clustering at individual and community levels.

© 2023 Shen YC et al. *JAMA Health Forum.*

eTable 5. Risk-Adjusted Percentage Point Changes in Probability of Mental Health Visit When Psychiatrist Capacity Changes: Separate Estimate for Increased and Decreased Capacity

	Visit made in MTF	Visit made in civilian
Mean quarterly rate at reference group	7.08%	1.81%
**MTF psychiatrist capacity increases**		
no psychiatrist within 30-min	0.00	0.00
<1 psychiatrist per 20K	0.27**	-0.04
	[0.17,0.36]	[-0.09,0.01]
1-3 psychiatrist per 20K	1.19**	0.06
	[1.07,1.31]	[-0.00,0.12]
>3 psychiatrist per 20K	1.39**	-0.04
	[1.22,1.56]	[-0.12,0.04]
**MTF psychiatrist capacity decreases**		
no psychiatrist within 30-min	0.00	0.00
<1 psychiatrist per 20K	0.26**	0.18**
	[0.16,0.36]	[0.12,0.24]
1-3 psychiatrist per 20K	0.60**	-0.05
	[0.48,0.71]	[-0.11,0.02]
>3 psychiatrist per 20K	0.57**	-0.00
	[0.42,0.73]	[-0.08,0.08]
**Civilian psychiatrist capacity increases**		
no psychiatrist within 30-min	0.00	0.00
<1 psychiatrist per 20K	-1.02**	0.38**
	[-1.40,-0.64]	[0.23,0.54]
1-3 psychiatrist per 20K	-1.77**	0.54**
	[-2.18,-1.37]	[0.38,0.71]
>3 psychiatrist per 20K	-2.44**	0.62**
	[-2.90,-1.99]	[0.43,0.82]
**Civilian psychiatrist capacity decreases**		
no psychiatrist within 30-min	0.00	0.00
<1 psychiatrist per 20K	-1.30**	-0.00
	[-1.72,-0.88]	[-0.19,0.18]
1-3 psychiatrist per 20K	-1.77**	0.53**
	[-2.18,-1.36]	[0.36,0.70]
>3 psychiatrist per 20K	-2.68**	0.61**
	[-3.14,-2.23]	[0.42,0.80]
N	22,158,647	22,158,647

Note: * p<0.05 ** p<0.01. Models specifications and other control variables are identical to models in Table 2. Robust standard errors assume clustering at individual and community levels.

© 2023 Shen YC et al. *JAMA Health Forum.*
