# Supplementary material for: Propensity of US Military Personnel to Seek Mental Health Care When Community Psychiatric Capacity Changes
Source: JAMA Health Forum. 2023 Oct 6;4(10):e233330. doi: 10.1001/jamahealthforum.2023.3330 (PMC10559180; doi:10.1001/jamahealthforum.2023.3330)
Supplement: Supplement 2. — Data Sharing Statement [file jamahealthforum-e233330-s002.pdf]

Data Sharing Statement

Shen. Propensity of US Military Personnel to Seek Mental Health Care When Community Psychiatric Capacity Changes. *JAMA Health Forum*. Published October 06, 2023. doi:10.1001/jamahealthforum.2023.3330

Data
Data available: No

Additional Information
Explanation for why data not available: Data is proprietary and owned by the Defense Health Agency. We will provide a data dictionary upon request.
